# Impact of low lethal concentrations of buprofezin on biological traits and expression profile of chitin synthase 1 gene (*CHS1*) in melon aphid, *Aphis gossypii*

**DOI:** 10.1038/s41598-019-48199-w

**Published:** 2019-08-23

**Authors:** Farman Ullah, Hina Gul, Hafiz Kamran Yousaf, Wang Xiu, Ding Qian, Xiwu Gao, Kaleem Tariq, Peng Han, Nicolas Desneux, Dunlun Song

**Affiliations:** 1 Department of Entomology, College of Plant Protection, China Agricultural University, Beijing, 100193 China; 2 Department of Agriculture, Abdul Wali Khan University Mardan, Khyber Pakhtunkhwa, Pakistan; 3 Key Laboratory of Biogeography and Bioresource of Arid Land, Chinese Academy of Sciences, Urumqi, 830011 China; 4 Université Côte d’Azur, INRA, CNRS, UMR ISA, 06000 Nice, France

**Keywords:** Entomology, Transcription

## Abstract

Buprofezin, a chitin synthesis inhibitor that can be used for the control of hemipteran pests, especially melon aphid, *Aphis gossypii*. The impact of low lethal concentrations of buprofezin on the biological parameters and expression profile of *CHS1* gene were estimated for two successive generations of *A*. *gossypii*. The present result shows that the LC_15_ and LC_30_ of buprofezin significantly decreased the fecundity and longevity of both generations. Exposure of F_0_ individuals to both concentrations delay the developmental period in F_1_. Furthermore, the survival rate, intrinsic rate of increase (*r*), finite rate of increase (*λ*), and net reproductive rate (*R*_0_) were reduced significantly in progeny generation at both concentrations. However, the reduction in gross reproductive rate (*GRR*) was observed only at LC_30_. Although, the mean generation time (*T*) prolonged substantially at LC_30_. Additionally, expression of the *CHS1* gene was significantly increased in F_0_ adults. Significant increase in the relative abundance of *CHS1* mRNA transcript was also observed at the juvenile and adult stages of F_1_ generation following exposure to LC_15_ and LC_30_. Therefore, our results show that buprofezin could affect the biological traits by diminishing the chitin contents owing to the inhibition of chitin synthase activity in the succeeding generation of melon aphid.

## Introduction

The melon aphid, *Aphis gossypii* Glover (Hemiptera: Aphididae), is a cosmopolitan sap-sucking pest that infests plants of Cucurbitaceae family worldwide^1,2^. *A*. *gossypii* cause damage to plants through direct feeding by curling and deforming the young leaves and twigs^3^. Moreover, melon aphids affect plants indirectly by transmitting plant viruses such as Cucumber Mosaic Virus (CMV)^2^ and by secreting honeydew, which causes the growth of black sooty mold^4^. The melon aphid transmits 76 viral diseases across 900 known host plants^5^. Different tactics have been used to control *A*. *gossypii*, but still, pesticides application remains the primary tool of Integrated Pest Management (IPM) programs against this pest^6^. However, the widespread use of insecticides such as organophosphates, carbamates, pyrethroids, and neonicotinoids has led to the development of resistance in aphids throughout the world^7–9^. Notably, previous studies stated that *A*. *gossypii* show higher resistance against neonicotinoid insecticides^7,8^. The increased resistance of *A*. *gossypii* against imidacloprid has been documented in China^10^.

Chitin is a polymer of N-acetyl-b-D-glucosamine which are crucial for insects in maintaining shape, providing strength and protection^11,12^. Chitin biosynthesis pathway is catalyzed by two chitin synthase enzymes encoded by two genes, i.e., Chitin synthase 1 (*CHS1*) and chitin synthase 2 (*CHS2*) having a significant role in insect growth and development^11,13^. *CHS1* is expressed in the exoskeleton structures encoding the isoform of enzymes that are responsible for the catalysis of chitin production in the cuticle^14,15^. *CHS2* is mainly present in the midgut epithelial cells encoding enzymes to synthesize chitin in the insect midgut^16^. *CHS1* gene has been cloned in many insects such as brown citrus aphid, *Toxoptera citricida*^17^, soybean aphid, *Aphis glycines*^18^, *Bactrocera dorsalis*^19^, *Plutella xylostella*^20^, *Nilaparvata lugens*^21^ and *Locusta migratoria*^22^. Despite that, due to the lacking of the peritrophic membrane, *CHS2* was not present in some insect pests^12,17,18^. The molting process accompanied by the formation of the cuticle is crucial for insect growth; therefore, suppression of chitin biosynthesis gives us an ideal platform for insect control^17,18^.

Insect growth regulator^23^, a class of insecticide having low vertebrate toxicity with a unique mode of action from currently used broad-spectrum neurotoxic insecticides^24^. Many IGRs have shown high efficiency against various insect species, as it disrupts the molting process of insect pest during developmental stages^24^, e.g. in the orders of Hemiptera^25^, Lepidoptera^26^, and Diptera^27^. Buprofezin is a chitin synthesis inhibitor prepared by Nihon-Nohyaku and is widely used against several sucking pests with very low risk to the environment^25,28–32^. Buprofezin is considered to be safe for humans owing to the absence of chitin biosynthesis pathway in vertebrates^18^. It has also been stated harmless for the natural enemies under field contexts^32^. Comprehensive knowledge about the exact mode of action of buprofezin is still lacking. However, initially it suppresses the chitin biosynthesis during molting and causes immature death during cuticle shedding^33^. Moreover, it disturbs the oviposition, egg fertility, and reduces fecundity after adult females were treated^25,33^.

Owing to the misapplication of pesticides and presence of their residues after degradation in fields^34^, the exposure of arthropods to low concentrations of these chemicals frequently occurrs, resulting in sublethal effects causing various physiological and behavioral disruptions in surviving organisms^35^ such as life span^36^, developmental rate^37^, fecundity^38^, feeding behavior^39^ and also alter the insect population dynamics^40^. Furthermore, such exposure to pesticides may also cause transgenerational effects, i.e., indirectly affecting the descendants^41^. Comprehensive studies about the impact of low concentration of insecticides have great importance to increase their rational application against target pests^29,30^. Hence, several studies reported the effects of buprofezin at sublethal or low lethal concentrations on insect pests^25,28,29^. Studies of these potential sublethal effects in-depth would help to improve the IPM programs.

These sublethal effects are usually detrimental to exposed individuals^35^. However, several studies have reported stimulatory impact when exposed to low or sublethal concentrations^42–45^. The stimulatory effects (known as hormesis) is a phenomenon that is encouraged by low dose while inhibited by high dose exposure of insecticides^46^. Several studies reported these hormetic effects on insect pests following exposure to insecticides, e.g., low dose of imidacloprid cause hormesis effect in *Myzus persicae* (Sulzer)^47^ and *Aphis glycines* (Matsumura)^48^. Recently, transgenerational hormesis has been observed in *A*. *gossypii* when exposed to nitenpyram at low lethal and sublethal concentration^45^. Besides, previous studies have also shown the insecticide stimulatory effect in *A*. *gossypii* at low doses of pirimicarb and flonicamid^49^.

A two-sex life table is widely used for investigating multiple sublethal effects of insecticides on insects, as it allows us to gain comprehensive knowledge that could be underrated at the individual level^50–53^. In-depth knowledge about the impact of low lethal concentrations of buprofezin on the biology of *A*. *gossypii* is still lacking. To address these gaps, we use age-stage life table parameters to appraise the sublethal and transgenerational effects of buprofezin on biological characteristics of *A*. *gossypii*. Moreover, to gain potential knowledge on impact of buprofezin on *A*. *gossypii*, we analyzed the expression profile of chitin synthase 1 gene (*CHS1*) at low dose exposure.

## Results

### Toxicity of buprofezin on melon aphids

Buprofezin toxicity against *A*. *gossypii* was determined following 48 and 72 h exposure (Table 1). The estimated value of LC_15_ was 1.125 mg L^−1^ and LC_30_ was 2.888 mg L^−1^, while LC_50_ was 7.586 mg L^−1^ after 48 h exposure of buprofezin. Similarly, the LC_15_, LC_30,_ and LC_50_ were 0.435 mg L^−1^, 0.898 mg L^−1^ and 1.886 mg L^−1^ respectively after 72 h exposure. The toxicity of buprofezin was higher at 72 h exposure. The low lethal concentrations LC_15_ and LC_30_ were selected to evaluate the sublethal effects of the buprofezin on the life history traits and expression profile of chitin synthase 1 gene (*CHS1*) in melon aphid following 72 h exposure.

**Table 1:** Toxicity of buprofezin against *A*. *gossypii* after 48 and 72 h exposure. ^a^Standard error; ^b^Confidence limits; ^c^Chi-square values and degrees of freedom.

Treatments	Slope ± SE^a^	LC_15_ mg L^−1^ (95% CL^b^)	LC_30_ mg L^−1^ (95% CL^b^)	LC_50_ mg L^−1^ (95% CL^b^)	χ^2^ (df)^c^	*P*
Buprofezin (48 h)	1.251 ± 0.165	1.125 (0.748 to 1.504)	2.888 (2.231 to 3.804)	7.586 (5.493 to 12.232)	3.358 (16)	0.999
Buprofezin (72 h)	1.627 ± 0.149	0.435 (0.302 to 0.571)	0.898 (0.702 to 1.098)	1.886 (1.570 to 2.274)	11.113 (16)	0.802

### Sublethal effects of buprofezin on parental aphids (F_0_)

LC_15_ and LC_30_ concentrations of buprofezin have significant effects on parental *A*. *gossypii* following 72 h exposure. Both concentrations (LC_15_ and LC_30_) significantly decreased the longevity (*F* = 103.22; df = 2, 25; *P* < 0.001) and fecundity (*F* = 160.40; df = 2, 25; *P* < 0.001) of the exposed F_0_ population. Furthermore, LC_30_ concentration of buprofezin showed a stronger effect compared to LC_15_ and the control (Table 2).

**Table 2:** Mean ( ± SE) developmental times (d) of various life stages of F_1_ generation *A*. *gossypii* produced from parents (F_0_) and longevity (d) and fecundity (d) of F_0_ generation treated with LC_15_ and LC_30_ concentrations of buprofezin for 72 h compared to the untreated control.

Treatments	1^st^ instar	2^nd^ instar	3^rd^ instar	4^th^ instar	Pre-adult	Adult Longevity F_1_	Adult Fecundity F_1_	Adult Longevity F_0_	Adult Fecundity F_0_
Control	1.85 ± 0.07b	1.48 ± 0.07b	1.32 ± 0.06b	1.03 ± 0.02c	5.68 ± 0.06c	25.38 ± 0.68a	35.17 ± 0.23a	21.16 ± 0.61a	28.86 ± 0.82a
LC_15_	1.97 ± 0.05b	1.62 ± 0.06b	1.47 ± 0.07b	1.28 ± 0.05b	6.33 ± 0.07b	18.40 ± 0.48b	28.30 ± 0.81b	14.03 ± 0.61b	18.03 ± 0.84b
LC_30_	2.17 ± 0.04a	1.98 ± 0.06a	1.88 ± 0.07a	1.82 ± 0.07a	7.85 ± 0.05a	10.83 ± 0.55c	12.27 ± 0.25c	8.30 ± 0.67c	9.01 ± 0.66c

Different letters within the same column represent significant differences at *P* < 0.05 level (one-way ANOVA followed by Tukey HSD tests).

### Transgenerational sublethal effects of buprofezin on progeny aphids (F_1_)

Impact of low lethal concentrations of buprofezin on progeny *A*. *gossypii* (F_1_) were determined (Table 2). The mean longevity (*F* = 153.82; df = 2, 178; *P* < 0.001) and fecundity (*F* = 527.07; df = 2, 178; *P* < 0.001) of F_1_ generation significantly decreased for LC_30_ and LC_15_. Furthermore, after exposure of F_0_ individuals to the LC_30_ of buprofezin, the developmental period of 1^st^ instar (*F* = 7.58; df = 2, 178; *P* < 0.001) and 2^nd^ instar (*F* = 14.19; df = 2, 178; *P* < 0.001) of F_1_ individuals were significantly prolonged. Similarly the duration of 3^rd^ instar (*F* = 16.31; df = 2, 178; *P* < 0.001) and 4^th^ instar (*F* = 48.43; df = 2, 178; *P* < 0.001) also increased significantly at LC_30_ of buprofezin. The total duration of pre-adult period (*F* = 273.70; df = 2, 178; *P* < 0.001) significantly increased in the offspring of F_0_ generation after treated by both concentrations of buprofezin compared to the control.

**Figure 1:**
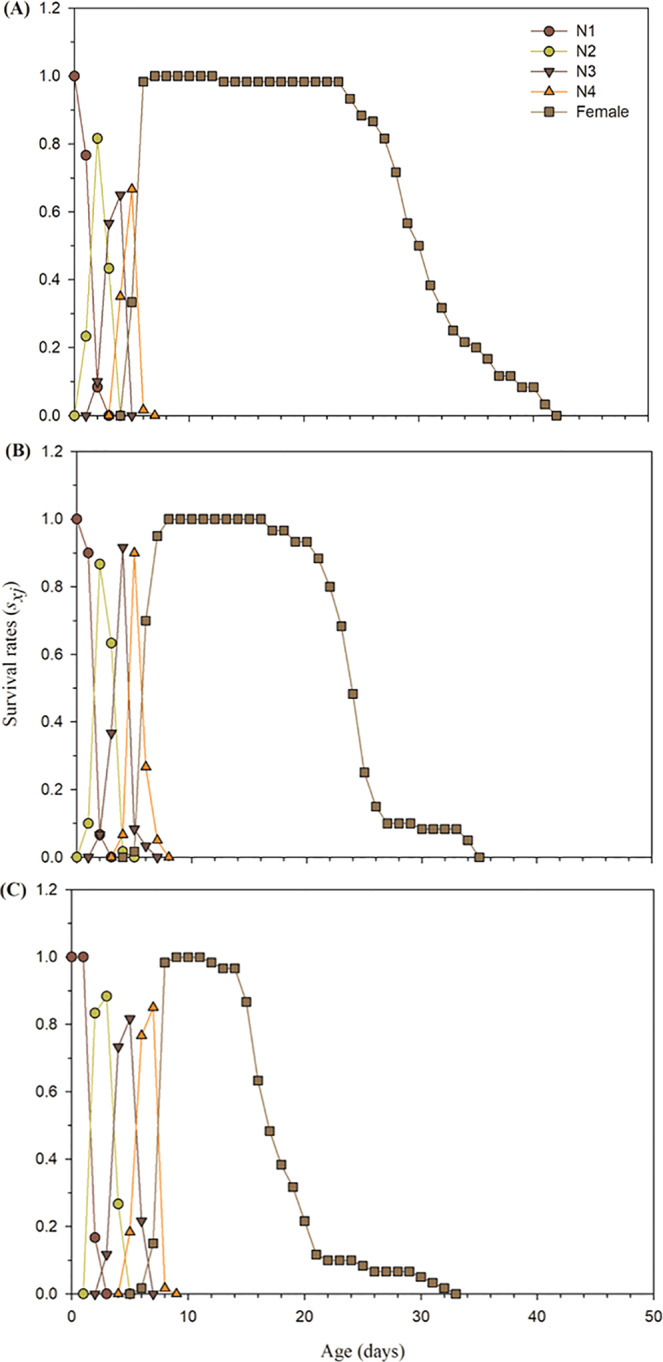
Age-stage specific survival rate (*s*_*xj*_) of *A*. *gossypii* of the F_1_ generation produced from parents (F_0_) under control condition (**A**), treated with LC_15_ (**B**), and treated with LC_30_ (**C**) of buprofezin.

Paired bootstrap technique was applied to determine the transgenerational impact of buprofezin (LC_15_ and LC_30_) on population growth using TWOSEX MS chart program^54^. The population parameters of F_1_ individuals, such as *λ*, *r*, *R*_0,_ and *GRR* were reduced at LC_30_ concentration. Obvious increase was noted for the mean generation time (*T*) at LC_30_. However, no effects were observed for the LC_15_ of buprofezin (Table 3). The age-stage specific survival rate (*s*_*xj*_) curves indicated variations in the developmental rates occurring among juvenile stages. Moreover, overlapping between different immature stages were shown in control (Fig. 1A), LC_15_ (Fig. 1B) and LC_30_ concentration of buprofezin (Fig. 1C). The adult survival rates differed among the buprofezin treatments (LC_15_ and LC_30_) and the control. Furthermore, the declined survival rate of melon aphid adults for LC_30_ concentration was recorded at the 12^th^ day (Fig. 1C) and the 16^th^ day was recorded for LC_15_ concentration (Fig. 1B), while the decline survival rate of melon aphid adults for the control was recorded at the 23^rd^ day (Fig. 1A).

**Table 3:** Transgenerational effects of buprofezin on population parameters of the F_1_ generation of *A*. *gossypii* whose parents (F_0_ generation) were treated with LC_15_ and LC_30_ concentrations compared to untreated control. *r*: intrinsic rate of increase (d^−1^), *λ*: finite rate of increase (d^−1^), *R*_0_: net reproductive rate (offspring/individual), *T*: mean generation time (d), *GRR*: gross reproductive rate were calculated using 100,000 bootstraps resampling.

Parameters	Control (Mean ± SE)	LC_15_ (Mean ± SE)	*P* value	Control (Mean ± SE)	LC_30_ (Mean ± SE)	*P*-value
*r*	0.302 ± 0.0054a	0.275 ± 0.0050b	0.0002	0.302 ± 5.413a	0.196 ± 0.0030b	<0.001
*λ*	1.353 ± 0.0073a	1.317 ± 0.0066b	0.0002	1.353 ± 7.325a	1.217 ± 0.0037b	<0.001
*R*_0_	35.166 ± 0.302a	28.236 ± 0.820b	<0.001	35.166 ± 0.302a	12.266 ± 0.256b	<0.001
*T*	11.771 ± 0.217a	12.125 ± 0.212a	0.243	11.771 ± 0.217b	12.741 ± 0.223a	0.0019
*GRR*	42.412 ± 0.923a	40.529 ± 1.359a	0.221	42.412 ± 0.923a	21.137 ± 0.983b	<0.001

Different letters within the same row show significant differences between control and buprofezin concentration groups (at the *P* < 0.05 level, paired bootstrap test using TWOSEX MS chart program).

**Figure 2:**
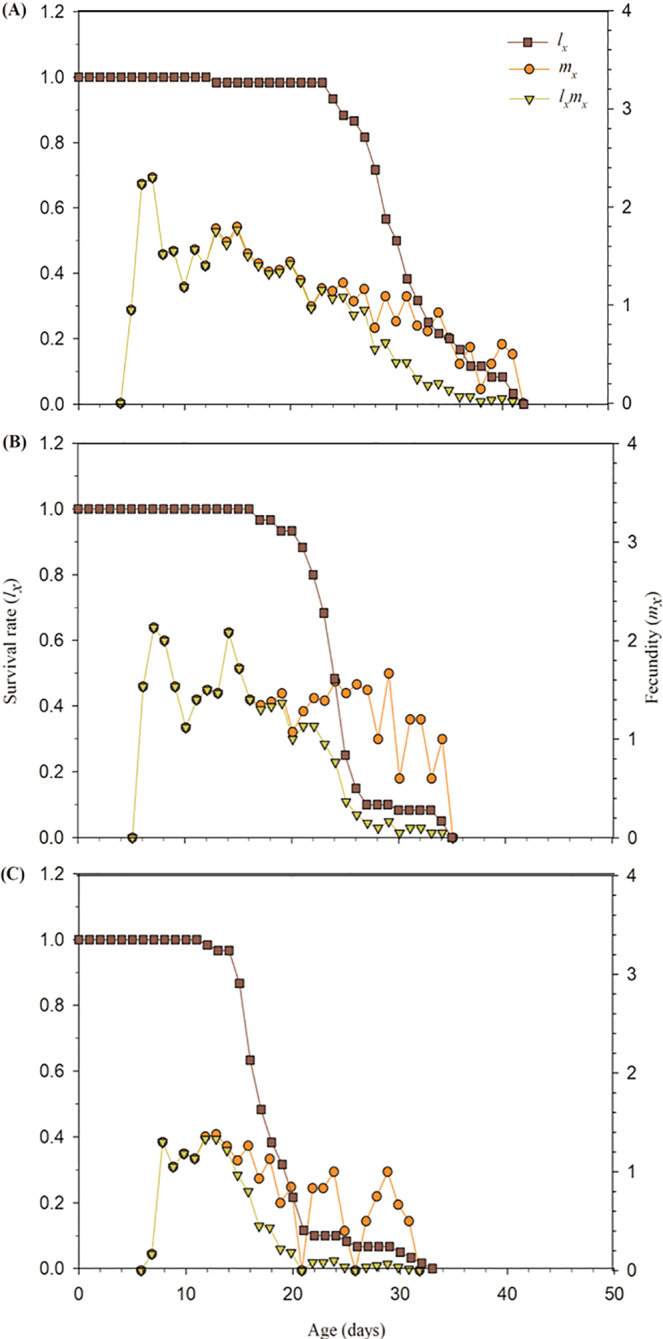
Age-specific survival rate (*l*_*x*_), age-specific fecundity (*m*_*x*_) and age-specific maternity (*l*_*x*_*m*_*x*_) of control (**A**) and *A*. *gossypii* individuals of F_1_ generation descending from F_0_ individuals exposed to the LC_15_ (**B**) and LC_30_ (**C**) concentrations of buprofezin.

The age-specific survival rate (*l*_*x*_), age-specific fecundity (*m*_*x*_), and the age-specific maternity (*l*_*x*_*m*_*x*_) for the treated and control *A*. *gossypii* are presented in Fig. 2. Compared to control treatment, the *l*_x_ value for LC_15_ and LC_30_ concentrations of buprofezin declined more rapidly. The population started to decrease after 23 days in control (Fig. 2A), whereas it declined after 16 days and 12 days in the LC_15_ and LC_30_ concentrations of buprofezin respectively (Fig. 2B,C).

The *m*_*x*_ and *l*_*x*_*m*_*x*_ values of the exposed *A*. *gossypii* were lower as compared to control (Fig. 2).

**Figure 3:**
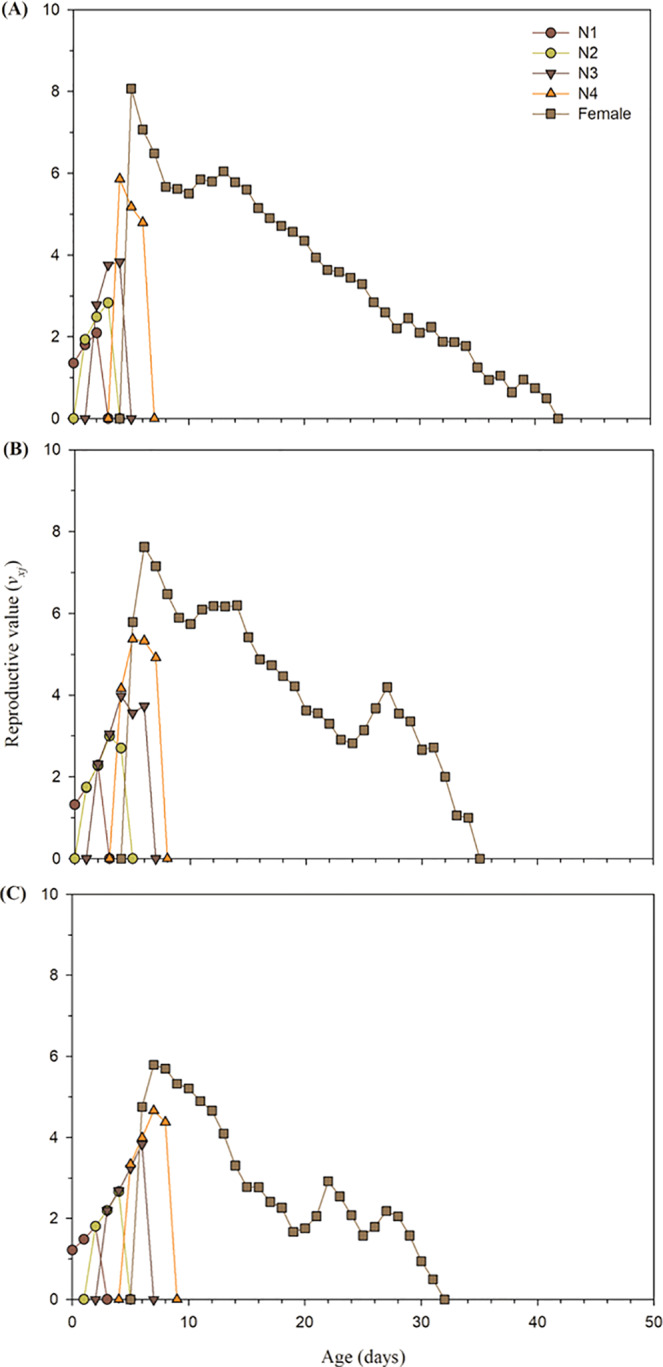
Age-stage reproductive value (*v*_*xj*_) of *A*. *gossypii* individuals of the F_1_ generation produced from parents (F_0_) under control conditions (**A**), treated with LC_15_ (**B**) and treated with LC_30_ (**C**) concentration of buprofezin.

**Figure 4:**
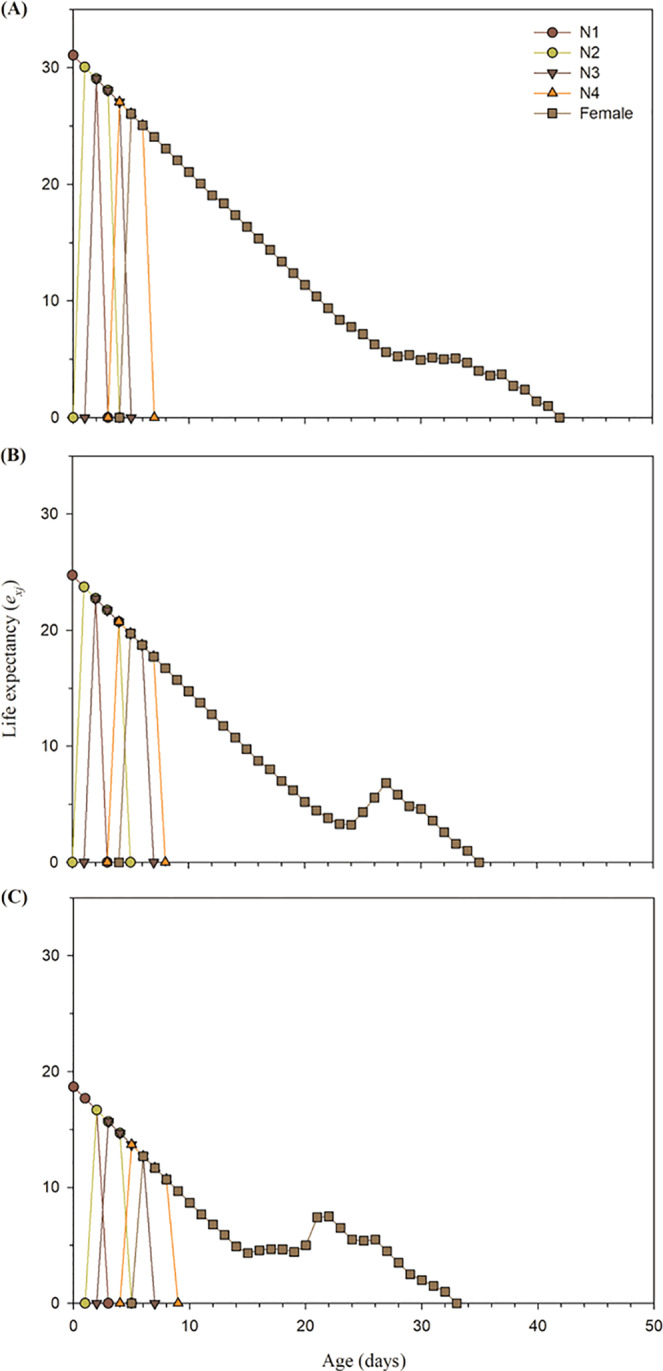
Age-stage specific survival rate (*e*_*xj*_) of *A*. *gossypii* descending from parents (F_0_) under control condition (**A**), treated with LC_15_ (**B**), and treated with LC_30_ (**C**) concentrations of buprofezin.

The age-stage reproductive values (*v*_*xj*_) of buprofezin treated adult aphids indicated that the *v*_*xj*_ of LC_15_ (Fig. 3B) and LC_30_ (Fig. 3C) concentrations of buprofezin was lower in contrast to the control individual (Fig. 3A). The *v*_*xj*_ value of LC_15_ (6.80 at the age of the 6^th^ day) and LC_30_ concentrations (4.9 at the age of the 7^th^ day) of buprofezin was lower compared to the control aphids (8 at the age of the 5^th^ day) (Fig. 3). Furthermore, the duration of F_1_ aphid’s reproduction was different after F_0_ generation exposure to buprofezin (LC_15_ and LC_30_) compared to the control. The *v*_*xj*_ value more than 4 was found for 17 days in the control group of melon aphid (Fig. 3A), while it was reported 14 and 7 days for LC_15_ and LC_30_ concentrations of buprofezin, respectively (Fig. 3B,C). The age-stage-specific life expectancy (*e*_*xj*_) of buprofezin treated *A*. *gossypii* (LC_15_ and LC_30_) was lower as compared to the untreated control group (Fig. 4).

### Sublethal effects of buprofezin on the expression profile of *CHS1* gene in melon aphid

**Figure 5:**
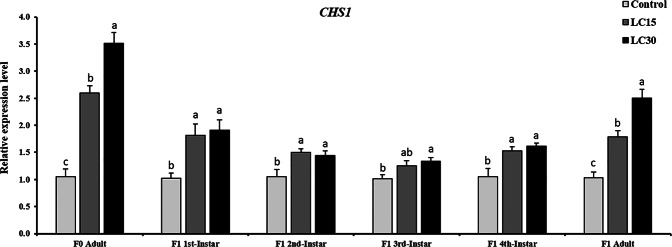
Relative expression levels of the chitin synthase 1 gene (*CHS1*) in F_0_ adults and in all developmental stages along with newly emerged adult melon aphid of F_1_ generation descending from the parent (F_0_) exposed to the LC_15_ and LC_30_ concentrations of buprofezin for 72 h. The relative expression level is expressed as the mean (±SE) with the control as the calibrator. Different letters above the error bars indicate significant differences at *P* < 0.05 level (one-way ANOVA followed by Tukey HSD tests).

Expression profile of *CHS1* gene in melon aphids was evaluated by quantitative real-time PCR (qPCR) during F_0_ adult and all developmental stages as well as newly emerged adult F_1_ individuals. The results indicated that the mRNA level was up-regulated in F_0_ adults and almost all stages of F_1_ melon aphids at LC_15_ and LC_30_ of buprofezin, after the exposure of parent generation (F_0_) for 72 h (Fig. 5). However, *CHS1* gene was relatively highly expressed in F_0_ adults treated with LC_30_ (3.51-fold), while it was 2.59-fold increase for the case of LC_15_ concentration of buprofezin. In the case of F_1_ generation descending from the treated parent (F_0_), *CHS1* gene was constantly expressed in aphids from 1^st^ instar to the newly emerged adult aphid. The mRNA level of *CHS1* was highly expressed in the 1^st^ instar (1.91-fold) and 2^nd^ instar nymph (1.44-fold) following exposure to buprofezin LC_30_. For LC_15_ - treated group, the relative expression was 1.81- and 1.50-fold for the 1^st^ and 2^nd^ instar nymph respectively, compared to the control. The *CHS1* gene was abundantly expressed 1.33-fold in 3^rd^ instar and 1.60-fold in the 4^th^ instar nymph after exposure to LC_30_, while they showed 1.24- and 1.53-fold increase for LC_15_ concentration of buprofezin. In F_1_ newly emerged adults, 2.50- and 1.78-fold abundance of the *CHS1* were observed at LC_30_ and LC_15_ concentrations of buprofezin, respectively (Fig. 5). The transcriptional level of *CHS1* gene increased 2.80- and 1.90-fold in parental aphids (F_0_) following 48 h exposure to the LC_30_ and LC_15_ concentrations of buprofezin respectively (Supplementary Fig. S1). While no effects were observed in the progeny generation (F_1_). No significant increase was noted for the *CHS1* gene transcription when melon aphids were treated to the two low lethal concentrations of buprofezin for 24 h (Supplementary Fig. S2).

## Discussion

The impact of buprofezin on the biological traits and expression profile of chitin synthase 1 (*CHS1*) gene of melon aphid were investigated following exposure to low lethal concentrations of this pesticide. Sublethal effects of buprofezin, e.g. reduced longevity and fertility have been reported in various insect pests, e.g. *Sogatella furcifera* Horvath (Hemiptera: Delphacidae)^29,30^, *Bemisia tabaci* (Hemiptera: Aleyrodidae)^25^, *Eretmocerus mundus* Mercet (Hymenoptera: Aphelinidae)^25^, *Encarsia inaron* (Hymenoptera: Aphelinidae)^28^. In this study, the impact of buprofezin at low lethal concentrations were examined demographically among two subsequent generations of *A*. *gossypii*. The results showed a decrease in longevity and fecundity of *A*. *gossypii* at LC_15_, and even more markedly at LC_30_ concentration of buprofezin in the progeny generation individuals. Similar effects were reported in the previous studies where the fecundity and longevity of *S*. *furcifera* females significantly reduced at sublethal doses of buprofezin^29,30^. The adult longevity and fecundity were decreased considerably when *A*. *gossypii* was treated to the LC_10_ and LC_40_ of cycloxaprid^55^. Additionally, low fertility has also been documented in *A*. *gossypii*^56^, *B*. *brassicae*^57^, and *Diaphorina citri*^58^ at sublethal concentrations of imidacloprid. Buprofezin inhibits the prostaglandin biosynthesis in *N*. *lugens* when treated to the sublethal concentrations resulting in spawning suppression^59^. Similar results have also been reported for a low dose of pyriproxyfen in *Aphis glycines* Matsumura (Hemiptera: Aphididae)^60^, *P*. *xylostella*^41^ and *Choristoneura rosaceana*^61^ (Lepidoptera: Tortricidae)^62^. These studies suggested that low lethal or sublethal concentrations of insecticides, including IGRs, adversely affect the longevity as well as the fecundity of exposed insect pests, which can be widely diffused in various IPM programs.

Transgenerational effects on the offspring of treated *A*. *gossypii* (F_0_) were also found. The longevity and fecundity of F_1_ individuals were decreased significantly at LC_15_ and LC_30_ concentrations, while the pre-adult period was increased. These effects are related to the reductions of F_1_ demographical parameters. We had shown the data that the demographical parameters, e.g. *r*, λ, and *R*_0_ were reduced significantly in F_1_ generation when its parents (F_0_) were subjected to LC_15_ and LC_30_ of buprofezin compared to the control; however, such negative impact on gross reproduction rate (*GRR*) was only evident at LC_30_ concentration of buprofezin. Previously, similar effects were documented on the offspring of white-backed planthopper, *Sogatella furcifera*^29^, cotton aphid, *A*. *gossypii*^63^, and brown planthopper, *Laodelphax striatellus*^64^ when subjected to the sublethal concentrations of buprofezin, sulfoxaflor, and thiamethoxam.

The analysis of the plotted curves for the *s*_*xj*_, *l*_*x*_, *m*_*x*_, *l*_*x*_*m*_*x*,_ and *e*_*xj*_ showed the adverse effects of buprofezin on the population growth parameters of *A*. *gossypii*. The *v*_*xj*_ stated that the reproduction duration of melon aphids was negatively affected when exposed to low doses buprofezin. The pre-adult period and mean generation time (*T*) were increased due to different physical and chemical processes when treated with buprofezin. Similar effects at the demographical level have been presented in various other reports^29,30,65,66^. Previous reports suggested that exposure to sublethal concentrations of buprofezin can suppress the population growth of *S*. *furcifera* via impact on their survival and reproduction^29,30^. Additionally, adverse effects at the demographical level have been reported in melon aphid at 25 and 100 ppm of cucurbitacin B^65^. Moreover, sublethal concentrations of imidacloprid and pirimicarb decreased the longevity and population growth of *A gossypii*^67^. Soybean aphid also showed been reduced population growth when they were exposed to sublethal concentrations of imidacloprid^48^.

Chitin synthase 1 (*CHS1*) is crucial for the chitin synthesis^11^, which has been studied in various insects including soybean aphid, *Aphis glycines*, and brown citrus aphid, *Toxoptera citricida*^17,18^. In this study, the relative transcript level of *CHS1* gene was up-regulated in the F_0_ adult and in all nymphal stages of F_1_ generation when exposed to LC_15_ and LC_30_ of buprofezin for 72 h. A previous study documented similar results for the white-backed planthopper, *Sogatella furcifera* (Horváth) (Hemiptera: Delphacidae) when exposed to the LC_10_ and LC_25_ (0.10 and 0.28 mg/L) of buprofezin^30^. Additionally, the diflubenzuron exposure in insects including *Anopheles quadrimaculatus* Say (Diptera: Culicidae), *Aphis glycines* Matsumura (Hemiptera: Aphididae), *Panonychus citri* McGregor (Acari: Tetranychidae), and *Toxoptera citricida* Kirkaldy (Hemiptera: Aphididae) resulted in increased expression of *CHS1* gene, which may be linked to increased mortality^17,18,68,69^. As the IGRs including buprofezin causes reduction in chitin content owing to the inhibition of chitin synthase activity in the exposed insect pests, which are translated into abortive molting, reduce longevity, decrease fecundity, and direct mortality^17,18,30,68,69^. In our investigations, the melon aphid population dynamics were reduced owing to the low lethal concentrations of buprofezin, suggesting their effectiveness against this insect pests. Moreover, the increasing abundance of *CHS1* mRNA transcript may result from a regulatory feedback mechanism that compensates the *CHS* enzyme activity inhibited by buprofezin. The compensation mechanism that is proposed through overexpression of *CHS1* gene translated into overproduction of the *CHS1* protein. However, owing to the buprofezin exposure, the potential overproduction of *CHS1* protein would be insufficient to maintain a vital level of *CHS* catalytic activity. Finally, the compensation mechanism failed to restore the enzymatic activity in the presence of buprofezin translated into reduced chitin production and causes the insect mortality.

In contrast to all these results, several studies documented no effect of insect growth regulator (e.g., diflubenzuron) on *CHS1* gene expression in *Drosophila melanogaster* and *Tribolium castaneum*^70,71^. Therefore, future work needed to understand the biological significance of *CHS1* gene comprehensively and as well as the relevant molecular mechanisms in the buprofezin exposed insects.

In conclusion, the LC_15_ and LC_30_ were used to understand the consequences of buprofezin on the biological traits and as well as their impact on the expression level of *CHS1* gene in *A*. *gosspii* for over two successive generations. Results indicated a significant reduction of parental aphid’s longevity and fertility when treated to the LC_15_ and LC_30_ of buprofezin. Moreover, both concentrations of buprofezin delay the aphid developmental stage and suppress the population growth of the progeny generation (F_1_). Also, the *CHS1* gene mRNA abundance was increased significantly at both concentrations following 72 h exposure. However, the effects observed from confined experimental scales may not translate into population effects under field contexts. Therefore, further investigation is necessary under field conditions to fully understand the potential of buprofezin’s integration into an optimized IPM strategy to control this insect pest.

## Materials and Methods

### Insects and insecticide

Melon aphid was originally collected from melon plants at Weifang District, Shandong Province, China. These insects were reared on fresh cucumber plants and were maintained under standard laboratory conditions with a temperature of 25 ± 1 °C, 70 ± 10% relative humidity (RH) and a 16:8 h light/dark photoperiod. Buprofezin with 97.4%, of active ingredient, was obtained from Jiangsu Anpon Electrochemical Co., Ltd. China.

### Toxicity of buprofezin against *A*phis *gossypii*

Toxicity of buprofezin was tested on *A*. *gossypii* using widely applied leaf dip bioassay procedure^48,66,72,73^. To ensure that all melon aphids were of same age and life instar, about 450 melon aphid adults were introduced on fresh cucumber plants. All adult aphids were removed after 24 hours while the offspring were allowed to grow on plants for eight days without any insecticide application. At this time, the newly-born nymphs passed all developmental stages and became apterous adults^65,66^.

The stock solution of buprofezin (active ingredient 97.4%) was prepared in acetone. The concentrations were further diluted with distilled water containing 0.05% (v/v) Triton X-100 to obtain six concentrations (8, 4, 2, 1, 0.5 and 0.25 mg L^−1^) for bioassays. Fresh leaf discs of cucumber plants were dipped for 15 s in the buprofezin solutions. After air drying, discs of cucumber plants were placed on agar bed (2%) in the 12-well tissue culture plate. Adult aphids were inoculated on the treated disc using a soft brush. The plates were covered with Parafilm (Chicago, USA). Each treatment has three replications, and 20–30 aphids per replicate were used in bioassay. Distilled water containing 0.05% (v/v) Triton X-100 was used as a control. All plates were placed in standard laboratory conditions with a temperature of 25 ± 1 °C, RH of 75% and a 16:8 h light/dark cycle (L:D). After 48 and 72 h exposures, aphid’s mortality was checked. Aphids were considered dead if not show any movement after pushing gently^48,74^. Mortality of controlled aphids was less than 10%. PoloPlus 2.00 was used to determine the LC_15_, LC_30_ and LC_50_ of buprofezin.

### Sublethal effects of buprofezin on F_0_ melon aphid

The life history traits of parental *A*. *gossypii* (F_0_) were investigated following the previously described methods with slight modifications^65,66^. The stock solution of buprofezin was prepared using acetone. The tested concentrations of buprofezin (LC_15_ and LC_30_) was prepared in distilled water containing 0.05% triton X-100. The low lethal concentrations of buprofezin (LC_15_ and LC_30_) were selected to determine their impact on melon aphids, as most of the pesticides were degraded after initial application by various factors^34,75^. Insecticide exposure was carried out, as discussed above. After 72 h exposure, sixty live and healthy aphids were collected from buprofezin treatments (LC_15_, LC_30)_ and control. The apterous melon aphid adults collected from LC_15_, LC_30_ and control were inoculated on fresh leaf discs individually^65,66^. Placed the treated discs on agar bed (2%) in the 12-well tissue culture plate. Parafilm (Chicago, USA) was used to cover the plate to prevent aphids escape. Fresh leaf discs were replaced throughout the experiment at every 3^rd^ day. All plates from buprofezin treatments (LC_15_, LC_30_) and control were placed under laboratory conditions as mentioned above. Longevity, as well as fecundity of *A*. *gossypii*, were noted daily till death.

### Transgenerational effects of buprofezin on F_1_*Aphis gossypii*

Impact of buprofezin at low lethal concentrations on the progeny generation (F_1_) of melon aphids were evaluated using the same method and treatments as discussed previously. The newly-born nymphs were individually retained on each insecticide-free cucumber leaf disc, and they were used as F_1_ generation of melon aphid. Sixty aphids were used for each of the treatment (LC_15_, LC_30_) and control. Each aphid was considered as a single replication. The number of offspring were counted on a daily basis until the death of the adults.

### Impact of buprofezin on chitin synthase 1 gene expression at low lethal concentrations in melon aphid

Impact of buprofezin exposure on chitin synthase 1 gene expression in melon aphid was evaluated using the same experimental setup as described above. Survived healthy melon aphid adults were collected after 24, 48, and 72 h exposure and stored in −80 °C as F_0_ generation. For F_1_ generation, exposed aphids collected from LC_15_, LC_30_ and control were transferred to new 20 mm diameter insecticide-free cucumber leaf discs. Aphids were collected at 4 developmental and newly emerged adult stages from both buprofezin treatments and control representing F_1_ generation. Total RNA was isolated from the exposed *A*. *gossypii* using TRIzol® reagent (Invitrogen, Carlsbad, CA, USA) following the manufacturer’s instruction. NAS-99 spectrophotometer (ACTGene) was used to analyze the RNA purity. Total RNA (1 μg) was used to synthesize the cDNA using the PrimeScript® RT Reagent Kit with the gDNA Eraser (Takara, Dalian, China). Real-time qPCR was performed using the SYBR® Premix Ex Taq™ (Tli RNaseH Plus) (Takara, Dalian, China). Primers for qPCR were synthesized using PRIMER 3.0 (http://bioinfo.ut.ee/primer3-0.4.0/) based on the conserved sequence of Soybean aphid (*Aphis glycines*) *CH1* gene (GenBank No. JQ246352.1) (Table 4). Elongation factor 1 alpha (*EF1α*), beta-actin (*β-ACT*) and glyceraldehyde-3-phosphate dehydrogenase (GAPDH) was used as an internal reference genes^76^. The reaction volume for qPCR was 20 μL including 10 μL of the SYBR®Premix Ex Taq, 1 μL of cDNA template, 0.4 μL of each primer, 0.4 μL of ROX Reference Dye II, and 7.8 μL of RNase-free water. The thermal cycling condition was initiated at 95 °C for 30 s, followed by 40 cycles of 95 °C for 5 s and 60 °C for 34 s, and a dissociation stage at 95 °C for 15 s, 60 °C for 1 min and 95 °C for 30 s, and 60 °C for 15 s. Three independent biological replicates with three technical replications were carried out for each qRT-PCR. To check the amplification efficiencies and cycle threshold (Ct), the standard curve was established with serial dilutions of cDNA (1, 1/10, 1/100, 1/1000, 1/10,000, and 1/100,000). Quantification of gene transcription was calculated using 2^−∆∆Ct^ method^77^.

**Table 4:** Primer sequences for chitin synthase 1 (*CHS1*) and internal control genes used to determine the expression profile in *A*. *gossypii* following exposure to buprofezin.

Primer name	Sequence (5′-3′)	Application
*CHS1*-F	ATTGCGTCACGATGATCCTT	qRT-PCR
*CHS1*-R	TGGTCGCTAGACGTTCACAC	qRT-PCR
EF1α-F	GAAGCCTGGTATGGTTGTCGT	qRT-PCR
EF1α-R	GGGTGGGTTGTTCTTTGTG	qRT-PCR
β-Actin-F	GGGAGTCATGGTTGGTATGG	qRT-PCR
β-Actin-R	TCCATATCGTCCCAGTTGGT	qRT-PCR
GAPDH-F	AACAGTTTTTTGAGTGGCGGT	qRT-PCR
GAPDH-R	TGGTGTCAACTTGGATGCGTA	qRT-PCR

### Data analysis

The LC_15_, LC_30_ and LC_50_ of buprofezin were analyzed using a log-probit model^78^ as commonly used in various studies^42,48^. The demographical (*r*, *λ*, *R*_0_, *T*, and *GRR*) and basic life table parameters (*s*_*xj*_, *v*_*xj*_, *l*_*x*_, *m*_*x*_, *l*_*x*_*m*_*x*,_ and *e*_*xj*_) were calculated using TWOSEX-MSChart program^54,79,80^. The intrinsic rate of increase (*r*) is classified as the population growth rate when the time advances infinity and population attains the stable age-stage distribution. The population will rise at the rate of *e*^*r*^ per time unit. It was calculated using eq. 1:

1 $$\mathop{\sum }\limits_{x=0}^{\infty }{e}^{-r(x+1)}{l}_{x}{m}_{x}=1$$

The finite rate of increase (*λ*) is defined as the population growth rate as the time approaches infinity and population attains the stable age stage distribution. It was calculated using Eq. 2:

2 $$\mathop{\sum }\limits_{x=0}^{\infty }\,({\lambda }^{-(x+1)}\mathop{\sum }\limits_{j=1}^{m}{f}_{xj}{S}_{xj})=1$$

The net reproductive rate (*R*_0_) is classified as the total fecundity produce by a common insect pest during the whole life. It was calculated using Eq. 3:

3 $$\mathop{\sum }\limits_{x=0}^{\infty }{l}_{x}{m}_{x}={R}_{0}$$

The mean generation time (*T*) is the duration of time that is needed by a population to enhance to *R*_0_-fold of its size as time advances infinity and the population calms down to a persistent age-stage distribution. It was measured using Eq. 4:

4 $$T=\frac{\mathrm{ln}\,{R}_{0}}{r}$$

The gross reproduction rate (*GRR*) was measured using Eq. 5:

5 $$GRR=\mathop{\sum }\limits_{x=0}^{\infty }{m}_{x}$$

The age-specific survival rate (*l*_*x*_) was measured using Eq. 6:

6 $${l}_{x}=\mathop{\sum }\limits_{j=1}^{m}{S}_{xj}$$

where *m* is the number of stages. Age-specific fecundity (*m*_*x*_) was measured through Eq. 7:

7 $${m}_{x}=\frac{{\sum }_{j=1}^{m}{S}_{xj}\,{f}_{xj}}{{\sum }_{j=1}^{m}\,{S}_{xj}}$$

where *s*_*xj*_ is showing the expected survival rate of a newly-born nymph to age x and stage j. The *e*_*xj*_ of an insect of age *x* and stage *y* showing the expected time duration to live. It was measured by Eq. 8:

8 $${e}_{xj}=\mathop{\sum }\limits_{i=x}^{\infty }\,\mathop{\sum }\limits_{y=j}^{m}{S{\prime} }_{iy}$$

Where *s*′_*ij*_ is the probability of an insect of age *x* and stage *y* will endure to age *i* and stage *j*. The age-stage reproductive value (*v*_*xj*_) is classified as the expectation of an insect of age x and stage y to the future offspring. It was measured using Eq. 9:

9 $${V}_{xj}=\,\frac{{e}^{r(x+1)}}{{S}_{xj}}\mathop{\sum }\limits_{i=x}^{\infty }{e}^{-r(i+1)}\mathop{\sum }\limits_{y=j}^{m}S{\text{'}}_{iy}\,{f}_{iy}$$

Population parameters were calculated and compared through paired bootstrap test^81^ using TWOSEX-MSChart with 100,000 replicates^53,82^. The results related to fecundity, longevity, developmental periods and *CHS1* expression of *A*. *gossypii* were calculated by One-way analysis of variance (ANOVA) with Tukey post hoc test (*P* < 0.05) (IBM, SPSS Statistics).

## Supplementary information

Supplementary Materials

Supplementary Table S1

Supplementary Table S2

Supplementary Table S3

Supplementary Table S4

Supplementary Table S5

Supplementary Table S6

## Data Availability

All data generated and analysed during this study are included in this published article (and its Supplementary Files). Supplementary Table S1: Lifetable of adult *A*. *gossypii* (F_0_ generation) following 72-h exposure to control solution. Supplementary Table S2: Lifetable of adult *A*. *gossypii* (F_0_ generation) following 72-h exposure to LC_15_ of buprofezin. Supplementary Table S3: Lifetable of adult *A*. *gossypii* (F_0_ generation) following 72-h exposure to LC_30_ of buprofezin. Supplementary Table S4: Lifetable of progeny generation (F_1_) produced by untreated adult *A*. *gossypii*. Supplementary Table S5: Lifetable of progeny generation (F_1_) produced by parental *A*. *gossypii* treated with LC_15_ of buprofezin. Supplementary Table S6: Lifetable of progeny generation (F_1_) produced by parental *A*. *gossypii* treated with LC_30_ of buprofezin.
