# Author Correction: Impact of low lethal concentrations of buprofezin on biological traits and expression profile of chitin synthase 1 gene (*CHS1*) in melon aphid, *Aphis gossypii*

**DOI:** 10.1038/s41598-020-74318-z

**Published:** 2020-10-20

**Authors:** Farman Ullah, Hina Gul, Hafiz Kamran Yousaf, Wang Xiu, Ding Qian, Xiwu Gao, Kaleem Tariq, Peng Han, Nicolas Desneux, Dunlun Song

**Affiliations:** 1 Department of Entomology, College of Plant Protection, China Agricultural University, Beijing, 100193 China; 2 Department of Agriculture, Abdul Wali Khan University Mardan, Khyber Pakhtunkhwa, Pakistan; 3 Key Laboratory of Biogeography and Bioresource of Arid Land, Chinese Academy of Sciences, Urumqi, 830011 China; 4 Université Côte d’Azur, INRA, CNRS, UMR ISA, 06000 Nice, France

Correction to: * Scientific Reports* 10.1038/s41598-019-48199-w, published online 23 August 2019

The original version of this Article contained errors.

In Table 1, in the penultimate column the incorrect symbol for Chi-square was used in the column heading.

“x^2^ (df)^c^”

now reads:

“χ^2^ (df)^c^”

Further, Table 3 contained an error. The standard errors for the control, LC_15_ and LC_30_ were incorrectly reported, due to mistranscription of the exponent values. As a result, the originally reported values were three orders of magnitude too high.

For the parameter *r*, the values for ‘Control (Mean ± SE)’, ‘LC_15_ (Mean ± SE)’ and ‘LC_30_ (Mean ± SE)’,

“0.302 ± 5.413a, 0.275 ± 5.022b and 0.196 ± 3.035b”

now read:

“0.302 ± 0.0054a, 0.275 ± 0.0050b and 0.196 ± 0.0030b”

For the parameter *λ*, the values for ‘Control (Mean ± SE)’, ‘LC_15_ (Mean ± SE)’ and ‘LC_30_ (Mean ± SE)’,

“1.353 ± 7.325a, 1.317 ± 6.617b and 1.217 ± 3.695b”

now read:

“1.353 ± 0.0073a, 1.317 ± 0.0066b and 1.217 ± 0.0037b”.

In the Materials and Methods section under subsection ‘Data Analysis’, Equations (8) and (9) were incorrectly reported. The authors used the notation provided in the original study describing these Equations. This study was subsequently corrected^1^, but although the correct Equations were used for calculations, the incorrect Equations were included in the original version of the Article.

Therefore, Equation (8),

$$ e_{xj} =  \mathop \sum \limits_{i = x}^{n} \mathop \sum \limits_{j = y}^{m} S^{\prime}_{ij} $$

now reads:

$$ e_{xj} =  \mathop \sum \limits_{i = x}^{\infty } \mathop \sum \limits_{y = j}^{m} S^{\prime}_{iy} $$

In addition, Equation (9),

$$ V_{xj} = \frac{{e^{{ - r\left( {x + 1} \right)}} }}{{S_{xy} }}\mathop \sum \limits_{i = x}^{n} e^{{ - r\left( {i + 1} \right)}} \mathop \sum \limits_{j = y}^{m} S^{\prime}_{ij} f_{ij} $$

now reads:

$$ V_{xj} = \frac{{e^{{r\left( {x + 1} \right)}} }}{{S_{xj} }}\mathop \sum \limits_{i = x}^{\infty } e^{{ - r\left( {i + 1} \right)}} \mathop \sum \limits_{y = j}^{m} S^{\prime}_{iy} f_{iy} $$

In the same subsection, under Equation (9):

“Population growth (mean and standard error) was calculated through paired bootstrap test^81^ using TWOSEX-MS Chart with 100,000 replicates^53,82^.”

now reads:

“Population parameters were calculated and compared through paired bootstrap test^81^ using TWOSEX-MSChart with 100,000 replicates^53,82^.”

References 78 and 79 were incomplete and were incorrectly listed as ‘Finney, D. (Cambridge University Press, London, 1971)’ and ‘Huang, Y. B. & Chi, H. The age-stage, two-sex life table with an offspring sex ratio dependent on female age (2011)’ respectively. The correct references are given below.

Finney, D. J. Probit Analysis. Cambridge University Press, Cambridge (1971).

Huang, Y. B. & Hsin Chi. The age-stage, two-sex life table with an offspring sex ratio dependent on female age. *Journal of Agriculture and Forestry* **60**(4), 337–345 (2011).

Furthermore, in the Supplementary Materials file, the title,

“Impact of low-dose buprofezin on the biological traits and expression profile of chitin synthase 1 gene (*CHS1*) in melon aphid, *Aphis gossypii*”

now reads:

“Impact of low lethal concentrations of buprofezin on biological traits and expression profile of chitin synthase 1 gene (*CHS1*) in melon aphid, *Aphis gossypii*”

These errors have now been corrected in the HTML and PDF versions of this Article, and in the accompanying Supplementary Materials.

## Supplementary information

Supplementary file1
