# Supplementary material for: Impact of low lethal concentrations of buprofezin on biological traits and expression profile of chitin synthase 1 gene (CHS1) in melon aphid, Aphis gossypii
Source: Sci Rep. 2019 Aug 23;9:12291. doi: 10.1038/s41598-019-48199-w (PMC6707215; doi:10.1038/s41598-019-48199-w)
Supplement: Supplementary file 1 — Supplementary Materials [file 41598_2019_48199_MOESM1_ESM.docx]

**Impact of low lethal concentrations of buprofezin on biological traits and expression profile of chitin synthase 1 gene (*CHS1*) in melon aphid, *Aphis gossypii***

**Farman Ullah^1^, Hina Gul^1^, Hafiz Kamran Yousaf^1^, Wang Xiu^1^, Ding Qian^1^, Xiwu Gao^1^, Kaleem Tariq^2^, Peng Han^3^, Nicolas Desneux^4^ and Dunlun Song^1*^**

^1^ Department of Entomology, College of Plant Protection, China Agricultural University, Beijing 100193, China

^2^ Department of Agriculture, Abdul Wali Khan University Mardan, Khyber Pakhtunkhwa, Pakistan

^3^ Key Laboratory of Biogeography and Bioresource of Arid Land, Chinese Academy of Sciences, Urumqi, 830011, China

^4^ Université Côte d’Azur, INRA, CNRS, UMR ISA, 06000 Nice, France

Orcid for Farman Ullah: 0000-0001-6174-1425

Orcid for Kaleem Tariq: 0000-0002-0951-509X

Orcid for Xiwu Gao: 0000-0003-3854-2449

**Corresponding author:** Dunlun Song

**Email:** songdl@cau.edu.cn

**Supplementary Materials**

**
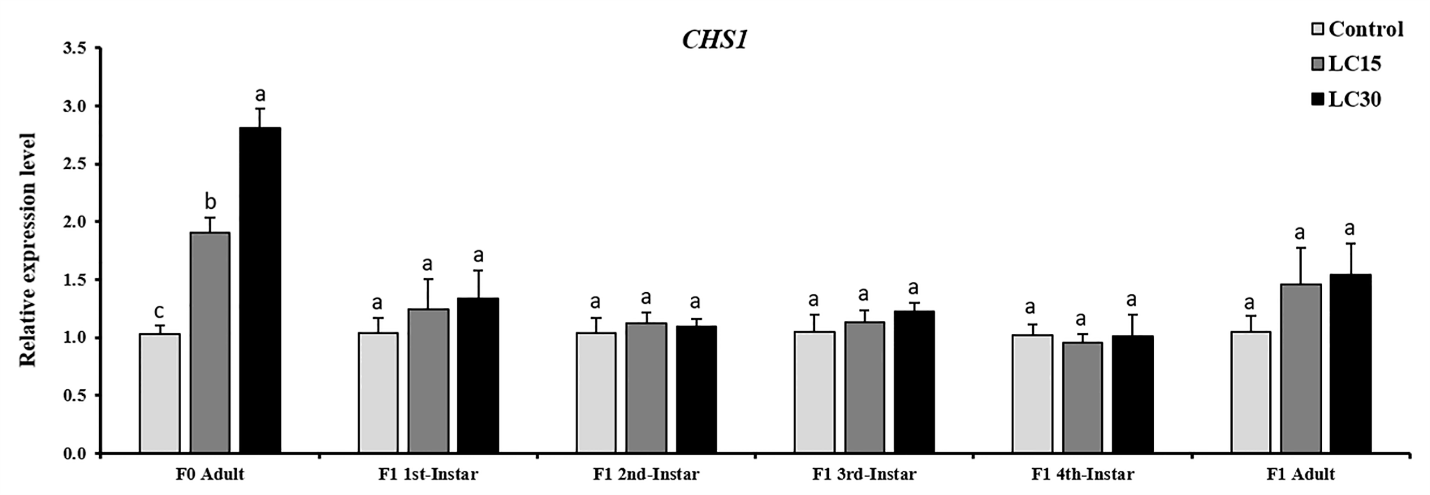
**

**Supplementary Figures 1.** Relative expression levels of the chitin synthase 1 gene (*CHS1*) in F_0_ adults and in all developmental stages along with newly emerged melon aphid adults of F_1_ generation descending from the parent (F_0_) exposed to the LC_15_ and LC_30_ concentrations of buprofezin for 48h. The relative expression level is expressed as the mean (±SE) with the control as the calibrator. Different letters above the error bars indicate significant differences at *P* < 0.05 level (one-way ANOVA followed by Tukey HSD tests).


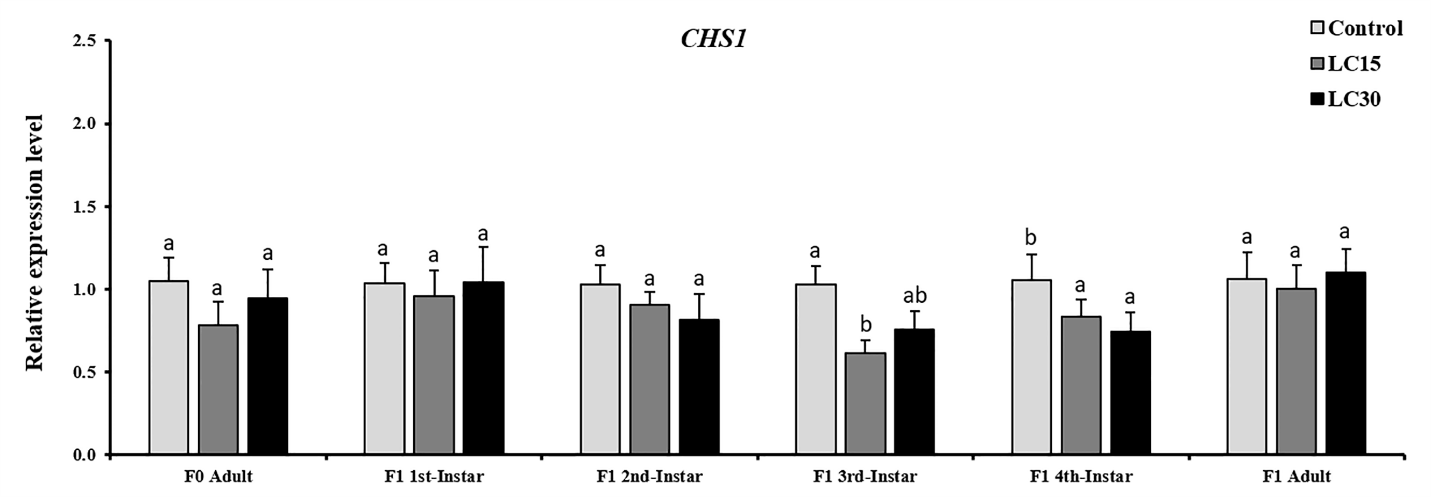


**Supplementary Figures 2.** Relative expression levels of the chitin synthase 1 gene (*CHS1*) in F_0_ adults and in all developmental stages along with newly emerged melon aphid adults of F_1_ generation descending from the parent (F_0_) exposed to the LC_15_ and LC_30_ concentrations of buprofezin for 24h. The relative expression level is expressed as the mean (±SE) with the control as the calibrator. Different letters above the error bars indicate significant differences at *P* < 0.05 level (one-way ANOVA followed by Tukey HSD tests).

**Supplementary Table Legends**

**Supplementary Table S1:** Lifetable of adult *A. gossypii* (F_0_ generation) following 72-h exposure to control solution

**Supplementary Table S2:** Lifetable of adult *A. gossypii* (F_0_ generation) following 72-h exposure to LC_15_ of buprofezin

**Supplementary Table S3:** Lifetable of adult *A. gossypii* (F_0_ generation) following 72-h exposure to LC_30_ of buprofezin

**Supplementary Table S4:** Lifetable of progeny generation (F_1_) produced by untreated adult *A. gossypii*

**Supplementary Table S5:** Lifetable of progeny generation (F_1_) produced by parental *A. gossypii* treated with LC_15_ of buprofezin

**Supplementary Table S6:** Lifetable of progeny generation (F_1_) produced by parental *A. gossypii* treated with LC_30_ of buprofezin
